# Poly-ADP-Ribosylation of Estrogen Receptor-Alpha by PARP1 Mediates Antiestrogen Resistance in Human Breast Cancer Cells

**DOI:** 10.3390/cancers11010043

**Published:** 2019-01-04

**Authors:** Nicholas Pulliam, Jessica Tang, Weini Wang, Fang Fang, Riddhi Sood, Heather M. O’Hagan, Kathy D. Miller, Robert Clarke, Kenneth P. Nephew

**Affiliations:** 1 Molecular and Cellular Biochemistry Department, Indiana University, Bloomington, IN 47405, USA; npulliam@iu.edu; 2 Cell, Molecular and Cancer Biology Program, Medical Sciences Indiana University School of Medicine, Bloomington, IN 47405, USA; jesstang@indiana.edu (J.T.); wang654@iu.edu (W.W.); ffang@indiana.edu (F.F.); hmohagan@indiana.edu (H.M.O.); 3 Department of Biology, Indiana University, Bloomington, IN 47401, USA; soodr@indiana.edu; 4 Department of Medicine, Indiana University School of Medicine, Indianapolis, IN 46202, USA; kathmill@iu.edu; 5 Department of Oncology, Lombardi Comprehensive Cancer Center, Georgetown University, Washington, DC 20057, USA; clarker@georgetown.edu; 6 Department of Cellular and Integrative Physiology, Indiana University School of Medicine, Indianapolis, IN 46202, USA; 7 Department of Obstetrics and Gynecology, Indiana University School of Medicine, Indianapolis, IN 46202, USA

**Keywords:** breast cancer, estrogen receptor, tamoxifen, antiestrogen resistance, PARP inhibitor

## Abstract

Therapeutic targeting of estrogen receptor-α (ERα) by the anti-estrogen tamoxifen is standard of care for premenopausal breast cancer patients and remains a key component of treatment strategies for postmenopausal patients. While tamoxifen significantly increases overall survival, tamoxifen resistance remains a major limitation despite continued expression of ERα in resistant tumors. Previous reports have described increased oxidative stress in tamoxifen resistant versus sensitive breast cancer and a role for PARP1 in mediating oxidative damage repair. We hypothesized that PARP1 activity mediated tamoxifen resistance in ERα-positive breast cancer and that combining the antiestrogen tamoxifen with a PARP1 inhibitor (PARPi) would sensitize tamoxifen resistant cells to tamoxifen therapy. In tamoxifen-resistant vs. -sensitive breast cancer cells, oxidative stress and PARP1 overexpression were increased. Furthermore, differential PARylation of ERα was observed in tamoxifen-resistant versus -sensitive cells, and ERα PARylation was increased by tamoxifen treatment. Loss of ERα PARylation following treatment with a PARP inhibitor (talazoparib) augmented tamoxifen sensitivity and decreased localization of both ERα and PARP1 to ERα-target genes. Co-administration of talazoparib plus tamoxifen increased DNA damage accumulation and decreased cell survival in a dose-dependent manner. The ability of PARPi to overcome tamoxifen resistance was dependent on ERα, as lack of ERα-mediated estrogen signaling expression and showed no response to tamoxifen-PARPi treatment. These results correlate ERα PARylation with tamoxifen resistance and indicate a novel mechanism-based approach to overcome tamoxifen resistance in ER+ breast cancer.

## 1. Introduction

Estrogen receptor-α (ERα) is a nuclear receptor [1,2,3] that responds to estrogen stimulation by translocating to the nucleus and regulating the transcription of target genes, including ERα and genes involved in regulating tumor progression [4]. Nearly 70% of breast cancers express ERα and rely on estrogen binding for growth and promotion of tumorigenesis [4]. Many ERα+ breast cancer patients are treated with the anti-estrogen tamoxifen, which binds to the receptor within the ligand-binding domain (LBD). Mechanistically, binding of tamoxifen to the receptor results in a conformational change, initially decreasing the capacity of ERα to engage target genes. Aside from the tamoxifen-ERα interaction, tamoxifen-induced oxidative damage was reported to be an integral component of its therapeutic efficacy [5,6,7], through increased reactive oxygen species (ROS), and blocking ROS activity reduced anti-estrogen sensitivity [8]. Although highly effective in ER+ breast cancers, many of these patients develop resistance to tamoxifen, relapse and eventually succumb to the disease, despite continued ERα expression in their tumors [9]. In this regard, a better underlying mechanisms mediating tamoxifen resistance is of foremost clinical significance [10].

Endocrine resistance evolves from many mechanisms, including altered epigenetic regulation [11,12,13], as well as post-translational modifications (PTMs) of ERα [10,14,15]. Many PTMs modify the ability of ERα to interact with transcriptional cofactors and engage target gene sequences in response to tamoxifen therapy, shifting tamoxifen from an antagonist to an ERα agonist [15]. In this context, additional understanding of ERα regulation may help identify novel approaches to overcome tamoxifen resistance in the presence of persistent ERα expression.

Poly (ADP-ribose) polymerase 1 (PARP1) is a nuclear enzyme [16] that regulates cell signaling and the DNA damage response by poly-ADP-ribosylating (PARylating) target proteins, including PARP1 itself [17,18]. A critical role for PARP1 in tumor progression through regulation of oxidative DNA damage has been demonstrated and indicated as a mechanism to overcome PARPi resistance [19,20]. Classically, clinical applications of PARP inhibitors (PARPi), such as talazoparib and veliparib, target BRCA-related cancers with defects in HR-mediated DNA repair pathways [21,22,23]. In addition to DNA damage, PARP1 regulates the activity of many nuclear receptors, including ERα [24]. It was revealed in a breast cancer model (MCF7, ERα-positive) that in response to estradiol, ERα and PARP1 co-localize to ERα-target genes to regulate their expression [25]. Furthermore, in vascular smooth muscle cells, ERα was demonstrated to be PARylated by PARP1 within the DNA binding domain (DBD), allowing for increased and maintained nuclear localization of ERα [26]. However, whether or not ERα is PARylated in breast cancer cells, and if PARylation correlates with tamoxifen response, is unknown.

Based on previous reports demonstrating elevated oxidative stress in ERα-positive, tamoxifen resistant breast cancer [5,8,27] and the role of PARP1 in responding to oxidative damage [28,29], we hypothesized that combining the antiestrogen tamoxifen with a PARPi would sensitize tamoxifen resistant, ERα-positive breast cancer to tamoxifen therapy. We tested our hypothesis in a MCF7-based breast cancer cell model systems. Combining a PARPi with tamoxifen altered ERα PARylation and decreased ERα-PARylation, and this was sufficient to overcome tamoxifen resistance in hormone-refractory, ERα-positive breast cancer cells. Our results demonstrate a novel mechanism of resistance to antiestrogen therapy and support a therapeutic approach for overcoming endocrine-resistant, ERα-positive breast cancer.

## 2. Results

### 2.1. Survival of Tamoxifen-Sensitive and -Resistant Breast Cancer Cells Is Inhibited by Tamoxifen-Talazoparib Combination

**Figure 1:**
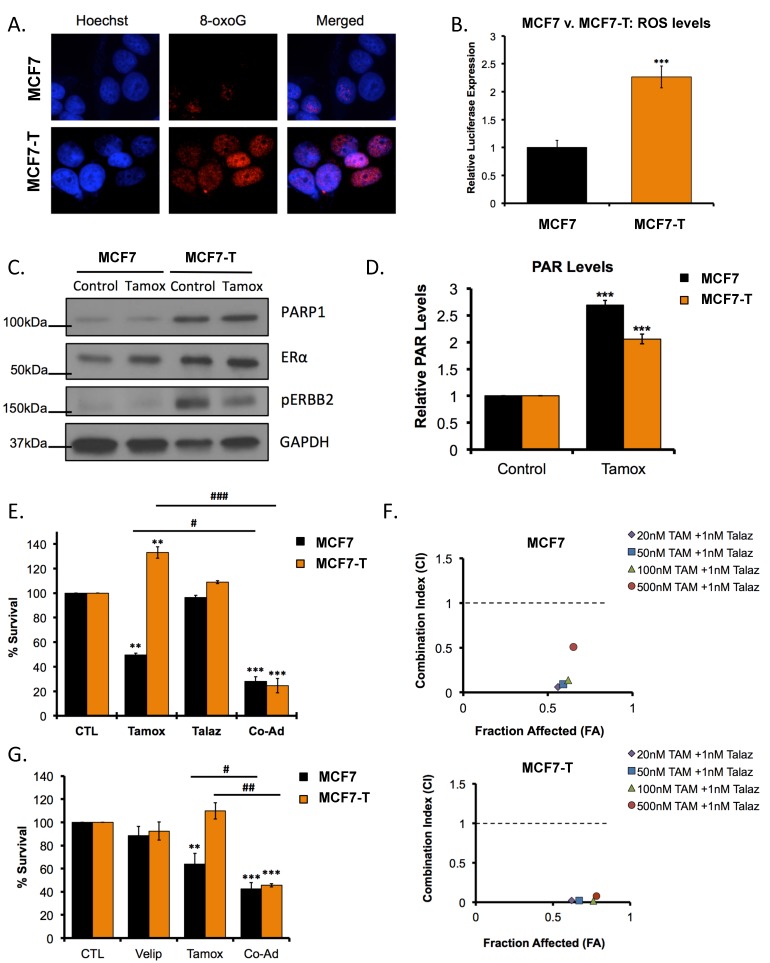
Therapeutic inhibition of PARP1 promotes sensitivity to tamoxifen treatment, in ER+ breast cancer, scale bar: 20 µm. (**A**) Immunofluorescence staining of 8-hydroxyguanosine (8-oxoG) in MCF7 and MCF7-T cell lines. (**B**) Basal ROS levels in MCF7 compared MCF7-T cells. Quantification is representative of at least three individual experiments. (**C**) MCF7 and MCF7-T cells were treated for 24 h with 100 nM tamoxifen (Tamox) and western blot analysis performed against the indicated antibodies. (**D**) MCF7 and MCF7-T cells were treated with Tamox (24 h, 100 nM) and subjected to PAR ELISA (**E**) MCF7 and MCF7-T cells were treated with 100 nM Tamox or 1 nM Talaz for 72 h, alone and in combination, and colony formation assay was performed. (**F**) MCF7 (Top) and MCF7-T (Bottom) cells were treated with Tamox and Talaz for 72 h, alone and in combination, and subjected to clonogenic survival assay to determine drug efficacy; x-axis is indicative of Fraction affected (FA), y-axis is indicative of the combination index (CI). Combinations beneath the black dashed line are synergistic. Results are representative of three independent experiments. (**G**) MCF7 and MCF7-T cells were treated with 100 nM Tamox or 10 nM veliparib (Velip) for 72 h, alone and in combination, and colony formation assay was performed. PAR, Poly (ADP-ribose). ** *p* < 0.001, *** *p* < 0.0001 compared to control, # *p* < 0.01, ## *p* < 0.001, ### *p* < 0.0001 relative to bracketed treatment.

We observed that tamoxifen-resistant (MCF7-T) cells showed increased levels of oxidative damage (Figure 1A), as indicated by immunofluorescence staining for the oxidative-damage marker 8-hydroxyguanosine (8-oxoG), consistent with previous studies [27] and validated this observation by measuring basal levels of reactive oxygen species (ROS) in tamoxifen-sensitive versus -resistant cell lines. As indicated by immunofluorescence, we observed increased (*p* < 0.05) levels of ROS in MCF7-T cells compared to the MCF7 parental cells (Figure 1B). We and others have demonstrated that oxidative damage caused by ROS promotes PARP1 activation [28,29]. We measured PARP1 levels and activity (indicated by poly-(ADP)-ribosylation (PARylation)), using western blot and ELISA assays and observed that basal PARP1 levels and activity were higher in MCF7-T cells compared to MCF7 cells (Figure 1C and Supplemental Figure S1A), as well as active ERBB2 (pERBB2), a marker of tamoxifen resistance [30]. Furthermore, tamoxifen treatment increased (*p* < 0.05) PARP1 activity in both parental and resistant cell lines (Figure 1D; ELISA assay).

To examine whether PARP1 inhibition altered cell sensitivity to tamoxifen, we treated MCF7 and MCF7-T cells with tamoxifen alone or in combination with talazoparib and performed colony formation assays. As expected, tamoxifen alone decreased (*p* < 0.05) MCF7 clonogenic survival, and increased (*p* < 0.05) MCF7-T cell clonogenicity (Figure 1E). Despite differential response to tamoxifen, co-administration of tamoxifen and talazoparib decreased (*p* < 0.05) cell survival in both MCF7 and MCF7-T cells (Figure 1E, Supplemental Figure S1B,C). The observed decrease in colony formation was synergistic (CI < 1) (Figure 1F, Supplemental Figure S1B,C), as determined by the Chou-Talalay method [31]. Similar combinatorial efficacy was observed upon co-administration of tamoxifen with the less potent PARPi veliparib (Velip; Figure 1G) [32].

To confirm the combinatorial efficacy of tamoxifen and talazoparib was not limited to the tamoxifen-resistant cells examined, we performed clonogenic survival assays in independently derived tamoxifen-resistant, ERα+ breast cancer cell lines (LCC2, LCC9; ref [33]). Treatment of LCC2 and LCC9 with tamoxifen-talazoparib decreased (*p* < 0.05) cell survival (CI < 1; Supplemental Figure S1D,E, respectively). Furthermore, PARP1 activity was increased (*p* < 0.05) in LCC2 and LCC9 cell lines compared to MCF7 parental cells (Supplemental Figure S1F) and tamoxifen further increased (*p* < 0.05) PARP1 activity (Supplemental Figure S1G).

To validate the observed decrease in colony formation by MCF7 and MCF7-T cells in anchorage-dependent growth conditions, survival was also measured under anchorage-independent conditions. Both MCF7 and MCF7-T cells were plated within an agarose substrate and treated with tamoxifen in the presence and absence of talazoparib. Consistently, tamoxifen alone decreased (*p* < 0.05) MCF7 cell survival, while combination tamoxifen-talazoparib further decreased (*p* < 0.05) both MCF7 and MCF7-T survival compared to control or either single agent (Supplemental Figure S2A).

### 2.2. Tamoxifen-Talazoparib Combinatorial Efficacy Is ERα-Dependent

To determine whether response to the combination was dependent on ERα, we treated MDA-MB-231 cells (TNBC; negative for ER, PR and HER2) and MCF7-F (clonally derived, fulvestrant resistant and lack ERα signaling; [30]) with tamoxifen in the presence and absence of PARPi, and performed colony formation assays. In response to tamoxifen alone, no effect on cell survival was observed in either cell line (Supplemental Figure S2B–D), as expected. Similarly, following co-administration of tamoxifen with PARPi, no increase in cell response to tamoxifen treatment was observed. Fulvestrant treatment with or without talazoparib had no effect on MCF7-F cell survival (Supplemental Figure S2C,D).

In response to tamoxifen treatment, ERα was shown to translocate to the nucleus in tamoxifen-resistant cells, with cytoplasmic localization being indicative of tamoxifen sensitivity [34]. In this regard, we observed tamoxifen treatment alone increased ERα nuclear localization (Supplemental Figure S3), consistent with previous reports [34], and co-administration with talazoparib increased both PARP1 and ERα cytoplasmic subcellular localization (caveolin-1 (Cav1) was used to validate decreased ERα function; [35]). Based on these results, it was next of interest to further examine a role for PARP1 in mediating tamoxifen resistance.

### 2.3. Tamoxifen-Talazoparib Co-Administration Increases Cellular DNA Damage

**Figure 2:**
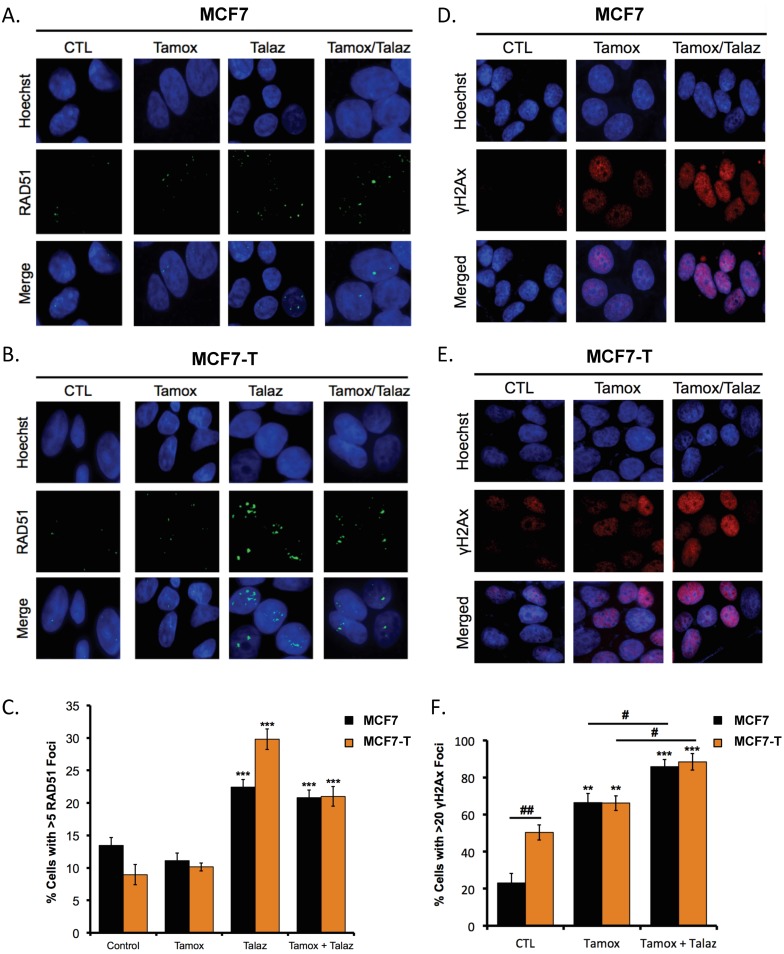
Tamoxifen-talazoparib co-administration induces DNA damage but not RAD51 foci formation. (**A**) MCF7 and (**B**) MCF7-T cells were treated with either 100 nM tamoxifen (Tamox) or 1 nM talazoparib (Talaz) for 72 h, alone and in combination. 24 h post treatment RAD51 foci formation assay was performed. Scale bar: 20 µm. (**C**) Quantification is representative of three individual experiments. (**D**) MCF7 and (**E**) MCF7-T cells were treated with 100 nM Tamox, with and without 1 nM Talaz for 48 h. 24 h post treatment cells immunofluorescence staining for γH2 AX was performed. Scale bar: 20 µm. (**F**) Quantification is representative of three independent experiments. * *p* < 0.001, *** *p* < 0.0001 compared to control, # *p* < 0.01, ## *p* < 0.001 relative to bracketed treatment.

Inhibition of PARP1 using clinical PARPi increases DNA double strand break (DSB) repair in BRCA-proficient cells, which can be assessed by RAD51 foci formation [36]. To examine whether altered DSB repair contributed to talazoparib-mediated tamoxifen sensitivity, we measured RAD51 foci formation in both MCF7 and MCF7-T cells following treatment with talazoparib in the presence and absence of tamoxifen. In response to talazoparib treatment alone, we observed increased (*p* < 0.05) RAD51 foci in both MCF7 and MCF7-T cell lines (Figure 2A–C), consistent with the synthetic lethal relationship between PARP and BRCA-mediated DNA damage repair [21,22]. Interestingly, tamoxifen treatment alone did not alter RAD51 foci formation compared to control, and tamoxifen-talazoparib co-administration had no effect on RAD51 foci formation compared to talazoparib alone (Figure 2A–C).

Because no specific effect on RAD51 was observed, we next examined DNA damage more generally. MCF7 and MCF7-T cells were treated with tamoxifen in the presence and absence of talazoparib, and immunofluorescence staining for γH2Ax foci formation was performed. In response to tamoxifen alone, we observed increased (*p* < 0.05) γH2Ax staining, which was further increased (*p* < 0.05) by co-administration with talazoparib (Figure 2D–F). These findings are consistent with a previous study by Qi et al. showing that tamoxifen can impair homologous recombination repair, while promoting DNA damage accumulation [37].

### 2.4. Tamoxifen Induces ROS, and ROS Promotes PARylaton of ERα

**Figure 3:**
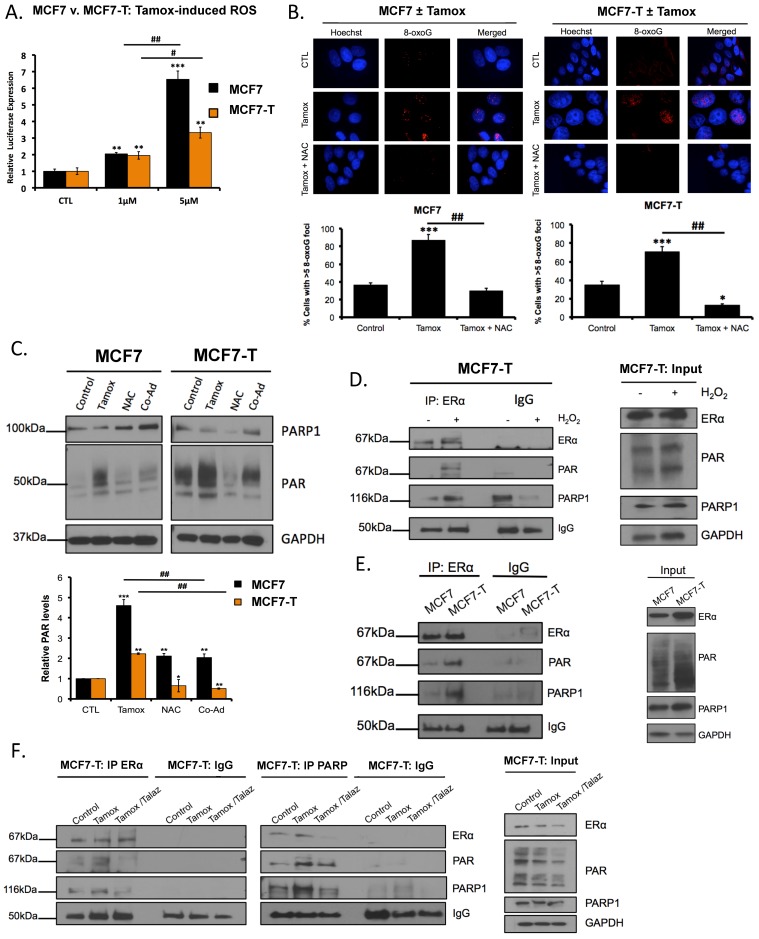
Tamoxifen-induced ROS accumulation promotes ERα PARylation. (**A**) MCF7 and MCF7-T cells were treated with the increasing concentrations of tamoxifen (Tamox) for 4 h, and subjected to ROS assay. (**B**) MCF7 and MCF7-T cells were treated with Tamox (1 μM) in the presence and absence of 1 mM *N*-acetyl-l-cysteine (NAC). 24 h post treatment immunofluorescence staining of 8-hydroxyguanosine (8-oxoG) was performed. Results are representative of three independent experiments, and quantified below. Scale bar: 20 µm. (**C**) MCF7 and MCF7-T cells were treated with 100 nM Tamox with or without 1 mM NAC (ROS scavenger). 24 h post treatment cell lysates were subjected to western blot analysis. (**D**) MCF7-T cells were treated with 2 mM H_2_O_2_ for 4 h. Treated cells were subjected to immunoprecipitation (ERα) and western blot analysis against the indicated antibodies. (**E**) MCF7 and MCF7-T subjected to immunoprecipitation (ERα) and western blot analysis against the indicated antibodies. (**F**) MCF7-T cells were treated for 4 h with 100 nM Tamox with or without 1 nM talazoparib (Talaz; pre-treat 24 h). Post treatment cells subjected to immunoprecipitation and western blot analysis against the indicated antibodies. * *p* < 0.01, ** *p* < 0.001, *** *p* < 0.0001 compared to control, # *p* < 0.01, ## *p* < 0.001 relative to bracketed treatment.

Because co-administration of tamoxifen and PARPi increased both tamoxifen response and γH2AX foci, but not RAD51 foci formation, it was of interest to further investigate the mechanism of PARPi-mediated tamoxifen sensitivity. We previously observed that basal ROS is increased in tamoxifen-resistant versus sensitive breast cancer cells (Figure 1A,B). Tamoxifen treatment increases intracellular ROS, and increased ROS is an important determinant of tamoxifen efficacy [5,8]. Based on these reports and our observations (Figure 2D–F), we tested whether tamoxifen increased oxidative damage and if tamoxifen-mediated ROS increased PARP1 activity. MCF7 and MCF7-T cells were treated with increasing concentrations of tamoxifen, and ROS levels were measured. We observed that tamoxifen increased (*p* < 0.05) ROS in a dose-dependent manner (Figure 3A), consistent with previous reports [5,8]. Furthermore, MCF7 and MCF7-T cells were treated with tamoxifen in the presence and absence of the ROS scavenger *N*-acetyl-l-cysteine (NAC) and cells were subjected to both immunofluorescence staining against 8-oxoG and western blot analysis. In response to tamoxifen, increased 8-oxoG staining was observed, which diminished upon co-administration with NAC (Figure 3B). Western blot analysis revealed increased PAR levels following tamoxifen treatment (Figure 3C and shown in Figure 1D), and co-administration with NAC decreased (*p* < 0.05) PAR levels (Figure 3C). Taken together, these data suggest tamoxifen increases PARP1 activity in a ROS-dependent manner.

Upon ROS-induced activation, PARP1 PARylates associated proteins to regulate cellular response to oxidative damage [28]. Moreover, in a vascular smooth muscle cell model [26], it was demonstrated that PARP1 PARylated ERα, resulting in altered ERα nuclear translocation, response to ligand and transcriptional activity. PARP1 activity has been shown to be an important regulator of ERα function [26]; however, whether ERα PARylation is involved in breast cancer and furthermore tamoxifen resistance has not been examined. To initially test if ERα was PARylated and whether ROS promoted ERα PARylation, we treated MCF7-T cells with H_2_O_2_ (2 mM for 3 h) and subsequently immunoprecipitated ERα. Following addition of H_2_O_2_ (oxidative damage conditions), we observed increased ERα PARylation and an increased ERα-PARP1 interaction (Figure 3D). To examine whether ERα was differentially PARylated in tamoxifen-sensitive versus -resistant breast cancer cells, ERα was immunoprecipitated in untreated MCF7 and MCF7-T cells, and PAR accumulation was examined by western blot analysis. Interestingly, we observed greater interaction between ERα and PAR in the tamoxifen-resistant MCF7-T cell line compared to the sensitive MCF7 cells (Figure 3E). These results demonstrate that oxidative damage is sufficient to promote ERα PARylation in breast cancer and correlate ERα PARylation with tamoxifen resistance.

### 2.5. Tamoxifen Promotes PARylation of ERα and PARylation Mediates ERα Response

We next hypothesized that tamoxifen-dependent ROS accumulation would likewise promote ERα PARylation. We treated both MCF7 and MCF7-T cells with tamoxifen, in the presence and absence of PARPi talazoparib and immunoprecipitated either ERα or PARP1 (MCF7-T cells). In response to tamoxifen treatment, we observed increased ERα PARylation in both MCF7 (Supplemental Figure S4A) and MCF7-T (Figure 3F) cells, and co-administration with talazoparib decreased the ERα PARylation, as well as the interaction with PARP1. Based on these results, we suggest that loss of ERα PARylation by PARPi treatment correlates with ERα response to ligands and increased cell sensitivity to antiestrogen therapy.

ERα is rapidly degraded in response to estradiol (E2) in breast cancer cells that are tamoxifen-responsive [30]. However, in tamoxifen resistant cells, E2 does not induce ERα degradation [30,33]. To validate that loss of PARP1 activity could mediate ERα response to ligand, we treated cells with E2 and performed western blot analysis. As a positive control, MCF7 cells were treated with E2 and reduced ERα levels were observed (Supplemental Figure S4B). In contrast, E2 did not decrease ERα protein levels in MCF7-T cells (Supplemental Figure S4C), whereas addition of the pure anti-estrogen fulvestrant markedly downregulated ERα protein levels (Supplemental Figure S4D). Next, MCF7-T cells were treated with talazoparib alone or in combination with E2, and under these conditions, a reduced level of ERα was observed (Supplemental Figure S4C). These results suggest that PARPi is able to regulate ERα response to ligand as a function of PARP1 activity, consistent with previous observations [25,26].

### 2.6. Talazoparib Reduces PARP1 and ERα Localization to ERα Target Genes

**Figure 4:**
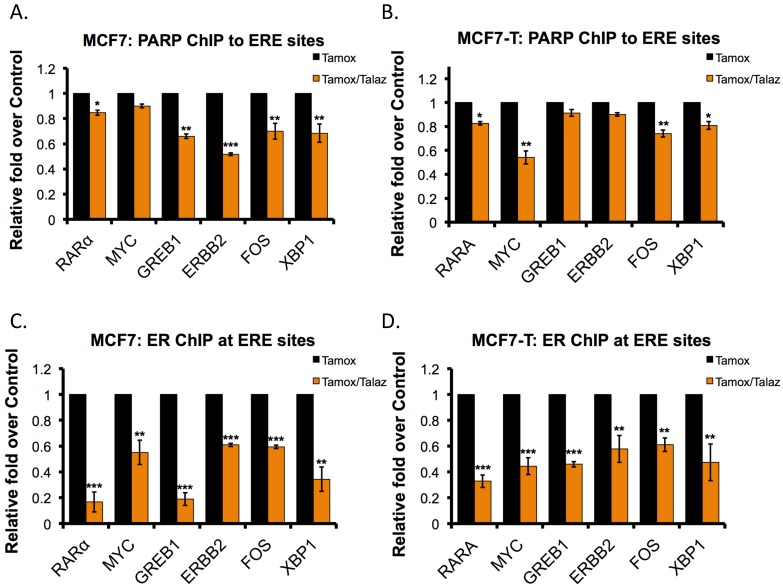
Talazoparib decreases PARP1 and ERα localization to ERα-target genes. Chromatin immunoprecipitation (ChIP) analysis of PARP1 localization to ERα-target genes (RARA, MYC, GREB1, ERBB2, FOS, XBP1) was performed in (**A**) MCF7 or (**B**) MCF7-T cells following tamoxifen treatment (Tamox; 100 nM) with or without talazoparib (Talaz; 1 nM). Chromatin immunoprecipitation (ChIP) analysis of ERα localization to ERα-target genes (RARA, MYC, GREB1, ERBB2, FOS, XBP1) was performed in (**C**) MCF7 or (**D**) MCF7-T cells following Tamox treatment (100 nM) with or without 1 nM Talaz. * *p* < 0.01, ** *p* < 0.001, *** *p* < 0.0001 compared to control.

Tamoxifen increased PARP1 activity, as well as ERα PARylation, and we observed that inhibition of PARP1 activity decreased nuclear ERα localization (Supplemental Figure S3). We then explored the role of PARP1 activity in regulating the enrichment of ERα at the promoter of ERα target genes. Based on ERα-binding sites of previously identified ERα target genes, described by Jin et al., we performed PARP1 chromatin immunoprecipitation (ChIP) analysis against a panel of genes (RARα, MYC, GREB1, ERBB2, FOS, and XBP1) [4]. As a positive control, we also performed ERα ChIP against the same gene panel. As predicted, treatment with talazoparib in both the tamoxifen-sensitive (MCF7) and -resistant (MCF7-T) cell lines resulted in decreased (*p* < 0.05) recruitment of PARP1 to ERα-binding sites in the majority of genes examined (Figure 4A,B). To a greater extent, ERα localization to ERα-binding sites was likewise decreased (*p* < 0.05) following tamoxifen-talazoparib treatment relative to tamoxifen treatment alone (Figure 4C,D). These results suggest that the ERα-PAR/PARP1 interaction is an important regulator of ERα-localization to ERα target genes.

### 2.7. Overexpression of miR-222 in Tamoxifen Resistant Cells Promotes PARP1 Activation

**Figure 5:**
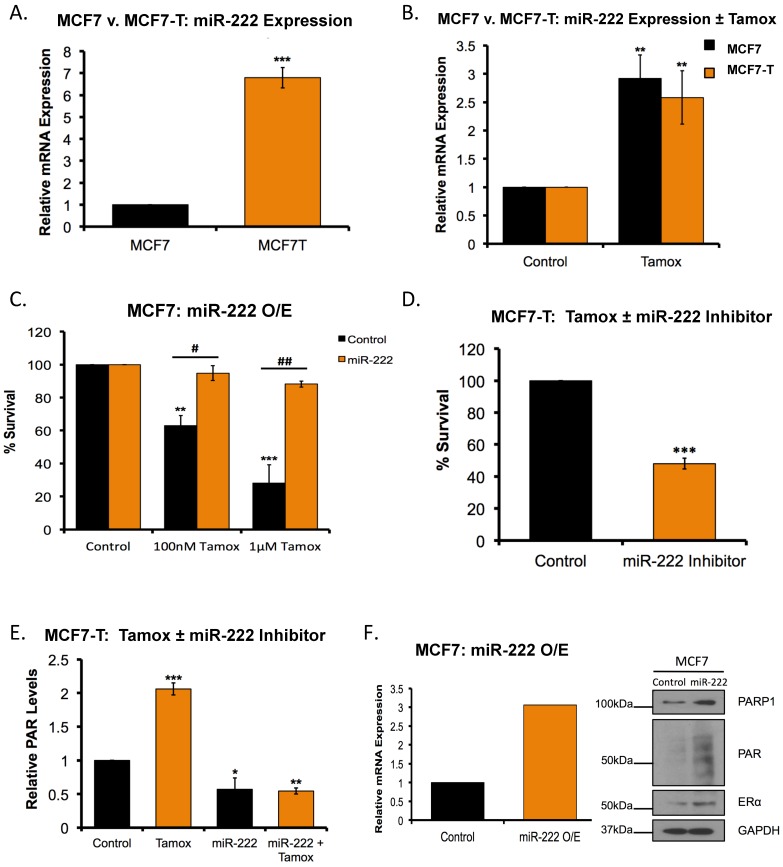
miR-222 mediates ER response to tamoxifen, and tamoxifen-mediated PARP1 activation. (**A**) Basal miR-222 expression was measured in MCF7 and MCF7-T cells by qRT-PCR analysis. (**B**) MCF7 and MCF7T cells were treated for 4 h with 100 nM tamoxifen (Tamox) and miR-222 expression was measured by qRT-PCR analysis. (**C**) MC7 cells were treated with the indicated concentration of Tamox for 4 h, with or without miR-222 overexpression. Treated cells were subjected to colony formation assays. Quantification is representative of three independent experiments. (**D**) MCF7-T cells were treated 72 h with 100 nM Tamox with or without miR-222 inhibition (KD). Treated cells were subjected to colony formation assays. Quantification is representative of three independent experiments. (**E**) MCF7-T cells were treated for 4 h with 100 nM Tamox with or without miR-222 inhibition. Post treatment cells were subjected to PAR ELISA. Quantification is representative of three independent experiments. (**F**) miR-222 was overexpressed in MCF7 cells and cell lysates subjected to qRT-PCR and western blot analysis. * *p* < 0.01, ** *p* < 0.001, *** *p* < 0.0001 compared to control, # *p* < 0.01, ## *p* < 0.001 relative to bracketed treatment.

While we demonstrated that tamoxifen increased ERα PARylation, we also observed that the basal ERα PARylation was increased in tamoxifen resistant versus sensitive cells (Figure 3E). As such, it was of interest to investigate the mechanism by which PARP1 was overexpressed in tamoxifen resistant breast cancer cells. It was recently demonstrated that microRNA-222 (miR-222) can both increase PARP1 activity, and promote PARPi sensitivity in ovarian cancer cells [38]. We and others demonstrated that miR-222 is overexpressed in tamoxifen resistant breast cancer cells compared to their sensitive counterparts [39,40,41]. To validate that miR-222 was overexpressed in tamoxifen resistant breast cancer, we measured basal miR-222 expression in parental MCF7 cells compared to MCF7-T by qRT-PCR analysis. We observed increased (*p* < 0.05; ~6.5-fold) miR-222 expression in tamoxifen resistant cells compared to the parental MCF7 cell line (Figure 5A). It was next of interest to determine whether tamoxifen could increase miR-222 expression and to test whether miR-222 overexpression was sufficient to promote tamoxifen resistance. MCF7 cells were treated with tamoxifen and qRT-PCR analysis was performed. As expected, tamoxifen treatment increased (*p* < 0.05) miR-222 expression in a dose-dependent manner (Figure 5B). We then treated MCF7 cells with tamoxifen in the presence and absence of miR-222 overexpression and subjected cells to clonogenic survival assays. In the absence of miR-222 overexpression, a dose-dependent decrease (*p* < 0.05) in cell survival was observed, and subsequent addition of miR-222 prevented tamoxifen-mediated cell death (Figure 5C). Consistently, we observed decreased (*p* < 0.05) cell survival in MCF7-T cells upon inhibition of miR-222 and treatment with tamoxifen (Supplemental Figure S5 and Figure 5D).

Given that tamoxifen was sufficient to increase both PARP1 activity and miR-222 expression, we determined whether tamoxifen-mediated PARP1 activation was miR-222 dependent. MCF7 cells were treated with tamoxifen in the presence and absence of miR-222 inhibitor and measured PAR levels. We observed that tamoxifen treatment increased (*p* < 0.05) PARP1 activity, as previously demonstrated, and subsequent inhibition of miR-222 decreased tamoxifen-mediated PARP1 activation (Figure 5E).

Regulation of PARP1 by miR-222 has been studied in depth in ovarian cancer cells [38]; however, whether miR-222 is sufficient to increase PARP1 activity in breast cancer is unknown. Using a miR-222 mimic, we exogenously expressed miR-222 in MCF7 tamoxifen-sensitive cells and measured PARP1 and PAR levels by western blot analysis. qRT-PCR validated overexpression of miR-222 (*p* < 0.05; ~3-fold) (Figure 5F). Furthermore, overexpression of miR-222 increased both PARP1 and PAR levels (Figure 5F).

### 2.8. miR-222 Regulates the Response to Tamoxifen-PARPi Co-Administration

**Figure 6:**
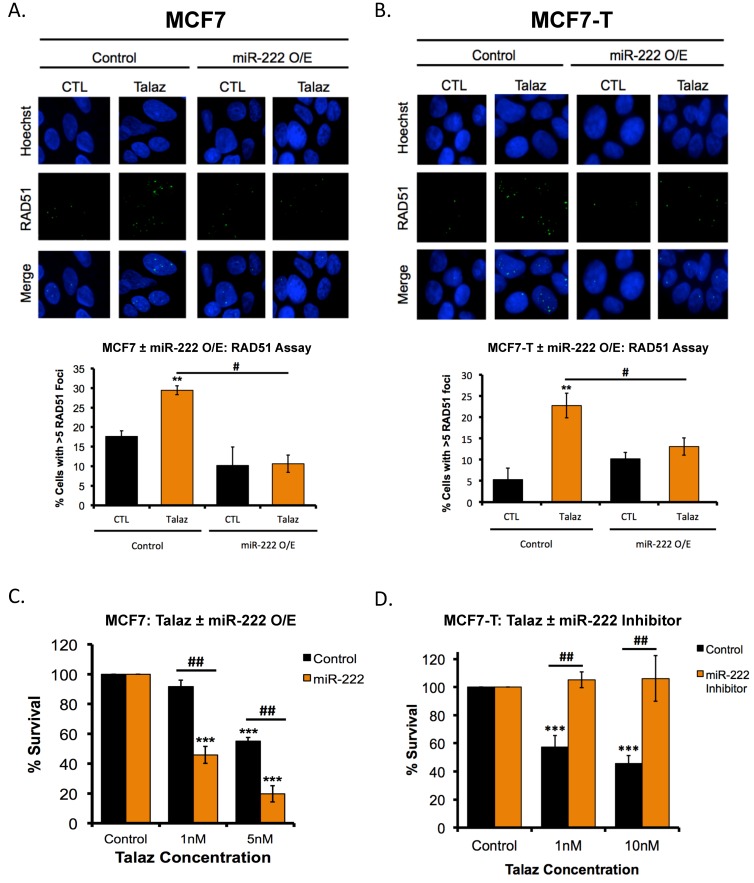
Tamoxifen-induced miR-222 expression mediates sensitivity to tamoxifen-talazoparib co-administration. (**A**) MCF7 and (**B**) MCF7-Tcells were treated with 1 nM talazoparib (Talaz) for 48 h in the presence or absence of miR-222 overexpression (O/E). Post treatment cells were subjected to RAD51 foci formation assay. Quantification is representative of three independent experiments. Scale bar: 20 µm. (**C**) MCF7 cells were treated with increasing concentrations of Talaz for 48 h, with or without miR-222 overexpression. Post treatment cells were subjected to colony formation assays. Quantification is representative of three independent experiments. (**D**) MCF7T cells were treated with 100 nM tamoxifen (Tamox) and increasing concentration of Talaz for 48 h, with or without miR-222 inhibition. Post treatment cells were subjected to colony formation assays. Quantification is representative of three independent experiments. ** *p* < 0.001, *** *p* < 0.0001 compared to control, # *p* < 0.01, ## *p* < 0.001 relative to bracketed treatment.

Because miR-222 overexpression increased PARP1 activity (Figure 5F), it was of interest to measure the effect on RAD51 foci formation. Consistent with our observation (Figure 2A–C), talazoparib treatment increased (*p* < 0.05) RAD51 foci in both MCF7 and MCF7-T cells (Figure 6A,B). Additionally, overexpression of miR-222 blocked formation of talazoparib-mediated RAD51 foci, irrespective of tamoxifen sensitivity (Figure 6A,B). We next investigated whether miR-222 mediated the PARPi response and, more importantly, the response to tamoxifen-talazoparib co-administration. First, we treated MCF7 cells with PARPi alone or combination with tamoxifen, in the presence and absence of miR-222 overexpression, and subjected cells to colony formation assays. In MCF7 cells overexpressing miR-222, we observed increased (*p* < 0.05) sensitivity to PARPi treatment (Figure 6C). Second, to determine whether miR-222 mediated the tamoxifen-talazoparib response, MCF7-T cells were treated with tamoxifen and increasing concentrations of talazoparib, with or without miR-222 expression. As miR-222 loss promoted sensitivity to tamoxifen (Figure 5D), tamoxifen-treated cells were used as the control (Figure 6D). In the presence of miR-222 (control), we observed a dose-dependent decrease in cell survival following tamoxifen-talazoparib co-administration; however, miR-222 inhibition diminished tamoxifen-talazoparib mediated cell death (Figure 6D). Taken together, our results support a role for talazoparib-mediated tamoxifen response in ER+, tamoxifen-resistant breast cancer.

## 3. Discussion

Most breast tumors express ERα, and stimulation of ERα by estradiol plays a significant role in breast tumor progression ERα-targeted therapies including tamoxifen demonstrate exceptional clinical efficacy in treating breast cancer [42]. However, due to long-term treatment, resistance to tamoxifen therapy is common, even though most resistant tumors continue to express ERα [4]. Mechanisms of resistance include epigenetic alterations [11,41] and post-translational modifications [14], which alter the antagonistic effects of tamoxifen on ERα function. Attempts to overcome tamoxifen resistance through development of novel therapies that target estrogen synthesis [15,43,44], and combination therapies have met with limited success, signifying a need to further explore therapeutic options to overcome tamoxifen resistance. In this regard, our study reveals that both oxidative damage and PARP1 levels are elevated in ERα+ tamoxifen resistant breast cancer. Addition of a PARPi increases cell response to tamoxifen therapy in an ERα-dependent manner. Through in vitro analysis, we show that combining tamoxifen with talazoparib increases both DNA damage and PAR accumulation and decreases survival of tamoxifen-resistant breast cancer cells. We identify miR-222 as a mediator of tamoxifen and PARPi response as well as the efficacy of tamoxifen-talazoparib combination. The results of this study support further investigation into combining antiestrogen with PARPi as a potential therapeutic option to overcome tamoxifen resistance in ERα+ breast cancer.

Elevated levels of oxidative stress in tamoxifen resistant breast cancer have been previously reported [27], which we postulate may correlate with increased PARP1 expression and activity, implicating PARP1 as a therapeutic target to overcome tamoxifen resistance [19,20]. With respect to our overall hypothesis, we demonstrate that intracellular ROS levels are elevated in tamoxifen resistant versus sensitive cells, as are PARP1 levels and activity. Based on overexpression of PARP1, we show co-administration of PARPi with tamoxifen decreases cell survival in ER+ breast cancer cells. However, in breast cancer cells that do not depend on estrogen signaling for the survival (MDA-MB-231, MCF7-F), the combination had no effect, demonstrating that ERα expression is integral in mediating tamoxifen-talazoparib induced cell death. Additionally, treatment with the PARPi alone increases DSB break repair, as indicated by increased RAD51 foci formation. However, tamoxifen alone or in combination with PARPi had no effect on RAD51 foci formation. An increase in γH2AX following tamoxifen and tamoxifen-talazoparib treatment further supports the notion that tamoxifen-talazoparib induces DNA damage.

In addition to inhibiting ERα function, tamoxifen-induced intracellular ROS contributes to its therapeutic efficacy [5]. Interestingly, increased oxidative damage has been hypothesized to act as a mechanism to overcome PARPi resistance and a distinct therapeutic vulnerability in cancer cells [19,45]. In support of these studies, we demonstrate that tamoxifen treatment increases ROS accumulation in both MCF7 and MCF7-T cell lines, and tamoxifen-mediated ROS accumulation is sufficient to promote PARP1 activation. Inversely, ROS depletion following tamoxifen treatment diminishes PARP1 activity. We posit that tamoxifen treatment results in PARP1 activation and ultimately PARPi-tamoxifen response in a ROS-dependent manner.

Our results relate ROS accumulation by tamoxifen to increase ERα PARylation. PARP1 regulates cell signaling, including DNA damage response, through rapid PARylation of target proteins, including PARP1 itself [24]. In vascular smooth muscle cells, ERα is directly PARylated by PARP1, altering the ability of ERα to bind its target genes [26]. To our knowledge, this is the first demonstration that ERα is PARylated in breast cancer cells and that tamoxifen can induce ERα PARylation. Moreover, treatment with talazoparib diminishes ERα PARylation, as well as the interaction between ERα and PARP1, which correlates with talazoparib-mediated tamoxifen response. Differential ERα PARylation in tamoxifen sensitive versus resistant cells correlates with tamoxifen sensitivity. As ERα functions as a nuclear receptor and a transcription factor, talazoparib treatment decreases both PARP1 and ERα localization to ERα-target genes, regardless of tamoxifen sensitivity. However, this observation is also consistent with decreased ERα levels following talazoparib treatment. Further experimentation will be necessary to understand the exact mechanism of talazoparib-mediated decrease in ERα localization to ERα-target genes.

In terms of a mediator of both tamoxifen and PARPi response, here we identify miR-222 as a potential negative regulator of tamoxifen-talazoparib mediated cell death. Tamoxifen resistant breast cancers overexpress miR-222 [39,40], and oxidative damage increases miR-222 expression. Interestingly, through inhibition of DSB damage repair, miR-222 overexpression is sufficient to promote PARPi sensitivity in ovarian cancer cells [38]. Beyond validating miR-222 overexpression in tamoxifen resistant cells, this is the first demonstration that upregulation of miR-222 increases PARP1 activation and PARPi sensitivity and decreases RAD51 foci formation in breast cancer cells. By inhibiting the oncomir (oncogenic miRNA), we further show the importance of miR-222 as a mediator of tamoxifen-talazoparib efficacy. Collectively, the data support a critical role for miR-222 in mediating response to tamoxifen-talazoparib treatment, with both loss and gain of miR-222 expression regulating response to single agent tamoxifen or talazoparib.

While the present study focuses on a proposed mechanism to overcome tamoxifen resistance through combination with PARPi, we do not believe this to be a mutually exclusive mechanism of induced cell death. In addition to PARPi-mediated tamoxifen response, our results also demonstrate a novel mechanism to overcome PARPi resistance, regardless of BRCA status, as all cell lines examined are BRCA-proficient and do not respond to PARPi as a single agent. Tamoxifen increases ROS and miR-222 expression but does not increase homologous recombination repair (RAD51 foci), all of which represent therapeutic avenues to increase sensitivity to PARPi [19,38,45], suggesting a novel feedback loop such that both tamoxifen increases sensitivity to PARPi and PARPi to tamoxifen.

Additionally, work by our group and Muvarak et al. demonstrate that addition of talazoparib to cells promotes PARP1 trapping to DNA, increases DNA damage and ultimately cell death in combination with DNMT inhibitors [29,46]. ROS induces several classes of DNA damage to which PARP1 may localize, and talazoparib is the most potent PARP1 trapper [32]. It is conceivable that tamoxifen-mediated ROS accumulation and co-administration with talazoparib promotes trapping of PARP1 to DNA, enhancing the efficacy of tamoxifen-talazoparib co-administration. In support of this possibility, we demonstrate that tamoxifen co-administration with the PARPi veliparib, a less potent PARP-trapper, was able to induce cell death but not to the extent of the tamoxifen-talazoparib combination treatment [32]. Moreover, we show tamoxifen-talazoparib co-administration reduces nuclear localization of both ERα and PARP1, though some PARP1 remains in the nucleus (Supplemental Figure S3). Talazoparib robustly reduces ERα at ERE sites and PARP1 but to a much lesser extent (Figure 4), indicating that PARP1 may be more tightly bound to the DNA (albeit not directly explored in this study). It is important to note that differences in ChIP enrichment at ERE sites may be due to either altered ERα and PARP1 nuclear localization, as demonstrated (Supplemental Figure S3), or decreased DNA affinity in the absence of PARP1 activity [27]. Further studies are needed to discriminate between these mechanisms. Nonetheless, it seems reasonable to suggest that some of the enhanced response to tamoxifen-talazoparib co-administration is a result of tamoxifen-mediated ROS accumulation and PARP1 trapping.

In summary, using a model system based on the MCF7 breast cancer cell line, we show addition of a PARPi increases cell response to tamoxifen in tamoxifen-resistant ERα+ breast cancer. We put forth a model of tamoxifen-mediated ROS accumulation, which increases miR-222 expression and subsequently PARP1 activity, priming the cell towards tamoxifen sensitivity, in an ERα-dependent manner (Supplemental Figure S6) and in the context of intact BRCA. Collectively, these data support additional pre-clinical and clinical investigation of tamoxifen-talazoparib combination for patients with tamoxifen resistant ERα+ breast cancer. Our future studies aimed at better understanding the significance of ERα PARylation and increasing the translational impact of the therapeutic combination will include multiple cell lines and in vivo model systems.

## 4. Materials and Methods

### 4.1. Cell Lines, Culture Conditions and Reagents

MCF7 and anti-estrogen resistant derivatives (MCF7-T and MCF7-F) were maintained and established as we have previously described [30]. Briefly, MCF7-T cells were maintained in tamoxifen-containing media, except prior to drug treatment. The maintenance concentration of tamoxifen was 100 nM. MCF7/LCC2 and MCF7/LCC9 cells lines (anti-estrogen resistant) were derived and maintained as described by Brünner et al. [33]. Cell lines were authenticated in 2017 by ATCC and tested for mycoplasma contamination (Manassas, VA, USA). To ensure cell line integrity, all cell lines were thawed at frequent intervals and not used beyond 30 passages. Additionally, cell morphology was monitored for each cell line, and proper media and growth conditions selected [29,30]. Hydrogen peroxide (H_2_O_2_) was purchased from EMD Millipore (Billerica, MA, USA). Tamoxifen (Tamox or 4-OHT) was purchased from Sigma Aldrich (St. Louis, MO, USA). Talazoparib (Talaz; PARPi) was provided by Pfizer/Medivation (San Francisco, CA, USA). Primary antibody dilutions were as followed: PARP1 (Cell Signaling, Danvers, MA, USA; 1:3000), PAR (Trevigen, Gaithersburg, MD, USA; 1:2000) ERα (Santa Cruz, Dallas, TX, USA; 1:2000), pERBB2 (Cell Signaling; 1:2000), GAPDH (Santa Cruz; 1:2000), IgG (Santa Cruz; 1:5000), β-Tubulin (Santa Cruz; 1:5000).

### 4.2. Clonogenic Survival and MTT Assay

Cells were seeded to 60–70% confluence in 10 cm plates and treated for the indicated times. 24 h following treatment, cells were washed, serially diluted, and plated in triplicate on 6-well plates (500–1000 cells/well). 6–14 days of cell growth were allowed for colony formation, followed by staining with 0.5% crystal violet. Cell counts were normalized to untreated controls as previously described [47]. Combination treatment scheme is as followed: Co-administration (‘Co-ad’) with both 100 nM tamoxifen (Tamox) and 1 nM talazoparib (Talaz) on day 1, and 100 nM Tamox on days 2 and 3, unless otherwise indicated.

### 4.3. RNA Extraction, Quantitative RT-PCR and Cell Transfection

RNA was extracted via RNeasy kit (Qaigen, Venlo, Limburg, The Netherlands), and cDNA prepared with MMLV reverse transcriptase (Promega, Madison, WI, USA). qRT-PCR was performed as we have previously described [48], using EEF1A as the internal control. Cells were treated with either miR-222 mimic (Ambion, Carlsbad, CA, USA; 4464066) or miR-222 inhibitor (Ambion; AM17000) within the indicated treatments conditions and according to the manufacturer’s protocol using the Lipofectamine 3000 reagent (Thermo Fisher Scientific, Waltham, MA, USA; L3000008).

### 4.4. PAR-Capture ELISA

Confluent (80%) 10 cm plates were treated as described in the text. Following treatment, samples were harvested, 20% SDS was added (to a final concentration of 1%), protein concentration measured according to the manufacturer’s protocol (Trevigen; 4520-096-K) and the lysate was snap-frozen and placed at −80 °C. Preparation of the PAR standard curve and treated samples was performed according to the manufacturer’s protocol, and as we have described [29]. In brief, 100 μL (20 μg protein) of each sample was added to a 96-well plate, pre-coated with PAR capture antibody and allowed to incubate overnight. Wells were then washed with PBST (PBS + 0.1% Tween 20) and incubated with primary antibody (PAR Polyclonal antibody), washed with PBST and incubated in secondary antibody (Goat Anti-Rabbit IgG HRP). Plates were washed with PBST, chemiluminescent substrate was added, and luminescence measured. The data are reported as the relative change in PAR concentration compared to control.

### 4.5. ROS Assays

Confluent 10 cm plates were treated as indicated in the text. Following treatment, 2 × 10^4^ cells/well were plated on 96-well plates. 24 h post plating, ROS assay, measuring H_2_O_2_ levels, (Promega) was performed as previously described [47].

### 4.6. Immunofluorescence

Cells were treated as described and plated on glass slides (50,000 cells/well), fixed and permeabilized as described by Sakai et al. [49]. Following permeabilization, cells were incubated for 1 h with RAD51 antibody (1:1000), γH2Ax antibody (1:1000), or 8-hydroxyguanosine (8-oxoG) (1:1000). Secondary antibody (Invitrogen, Carlsbad, CA, USA) was added and slides incubated for 45 min. Nuclei were counterstained with Hoechst stain (1:5000; 33342, Invitrogen). Images were captured using a Nikon NIE microscope, and processed using NIS Elements Viewer. Cells with 5 or more RAD51 foci were considered positive (per ref [49]) and cells with 20 or more γH2Ax foci were considered positive. Results are representative of three independent experiments ± SEM.

### 4.7. Western Blot Analysis

Total cell lysate was prepared with RIPA lysis buffer. Western blot analysis was performed as we have described previously [47].

### 4.8. Combination Index and Synergism

Cells were treated and plated as indicated for clonogenic survival assays (500 cells/well). Following treatment, percent survival subtracted from 100% was indicative of the fraction affected (FA). Subsequent combination indices, and synergism determination, were determined by the Chou-Talalay method ([31]; with mutually non-exclusive assumption) using CompuSyn Software [50].

### 4.9. Immunoprecipitation Assays

MCF7 and MCF7-T cells were grown to confluence (90%) in 15 cm plates, and treated as described in the text. 24 h following treatment, cells were washed with PBS and harvested in immunoprecipitation (IP) lysis buffer and protease inhibitors. The lysate was sonicated briefly and rotated at 4 °C, followed by centrifugation at 13,000 rpm for 10 min. The lysates were added to 50–60 μL of pre-conjugated protein A/G beads with the indicated antibodies. Beads were pelleted (1000 rpm, 4 °C) and washed in IP buffer. The samples were run on SDS-PAGE gel and immunoblot analysis was performed against the indicated antibodies [4,47].

### 4.10. Chromatin Immunoprecipitation (ChIP)-qPCR

MCF7 and MCF7-T cells were grown to confluency (90%) in 15 cm plates, and treated as described in the text. 24 h following treatment cells were washed with PBS, crosslinked with 10% formalin and harvested in immunoprecipitation (IP) lysis buffer. Following IP (previously described, [48]) with antibody pre-conjugated protein agarose beads, the crosslinks were reversed, and DNA was purified. ChIP-qPCR primers targeting the estrogen response element (ERE) were designed as previously described by Jin et al. [4]. A negative control region was used, as we have previously described [48].

### 4.11. Statistical Analysis

All data, unless noted otherwise, are represented as mean value ± SEM of at least three biological replicates. IC_50_ data was determined by Prism 6 (GraphPad Software, San Diego, CA, USA), using logarithm normalized sigmoidal dose-curve fitting. Tukey’s test for multiple comparisons correction was used to analyze the significance among different groups in biological assays, unless otherwise stated. For in vivo experiments, ANOVA/Mann-Whitney tests (GraphPad) were used to determine statistical significance, as described [46].

## 5. Conclusions

Therapeutic targeting of ERα by tamoxifen is standard of care for premenopausal breast cancer. While tamoxifen increases overall survival, tamoxifen resistance remains a major limitation, despite continued expression of ERα in resistant tumors. Towards a better molecular understanding of endocrine resistance, we used clonally derived ERα-positive, antiestrogen-resistant breast cancer MCF7-cell models and demonstrated differential ERα PARylation in tamoxifen-resistant vs. –sensitive cells. We further demonstrated that tamoxifen treatment was sufficient to activate PARP and increase ERα PARylation in an ROS-dependent manner. Combination treatment of the PARP inhibitor talazoparib with tamoxifen effectively blocked ERα PARylation, markedly decreased ERα localization to ERα target genes and significantly enhanced response to tamoxifen in an ERα-dependent manner in models of acquired tamoxifen resistance. We provide the first pre-clinical evidence that combining a PARP inhibitor with tamoxifen has significant activity against tamoxifen-resistant breast cancers which continue to express ERα, a therapeutic strategy that may warrant further investigation.

## Supplementary Materials

The following are available online at http://www.mdpi.com/2072-6694/11/1/43/s1, Figure S1: Tamoxifen-talazoparib co-administration decrease cell survival and increase PARP activity, Figure S2: Tamoxifen-talazoparib efficacy is ERα-dependent, Figure S3: Tamoxifen and talazoparib alter ERα nuclear localization, Figure S4: Tamoxifen induces ER PARylation in tamoxifen-sensitive MCF7 cell line, Figure S5: miR-222 inhibition decreases expression of miR-222, Figure S6: Proposed model.
