# Supplementary material for: Poly-ADP-Ribosylation of Estrogen Receptor-Alpha by PARP1 Mediates Antiestrogen Resistance in Human Breast Cancer Cells
Source: Cancers (Basel). 2019 Jan 4;11(1):43. doi: 10.3390/cancers11010043 (PMC6357000; doi:10.3390/cancers11010043)
Supplement: Supplementary file 1 [file cancers-11-00043-s001.pdf]

Supplementary Materials: Poly-ADP-Ribosylation of Estrogen Receptor-Alpha by PARP1 Mediates Antiestrogen Resistance in Human Breast Cancer Cells

Nicholas Pulliam, Jessica Tang, Weini Wang, Fang Fang, Riddhi Sood, Heather M. O'Hagan, Kathy D. Miller, Robert Clarke and Kenneth P. Nephew

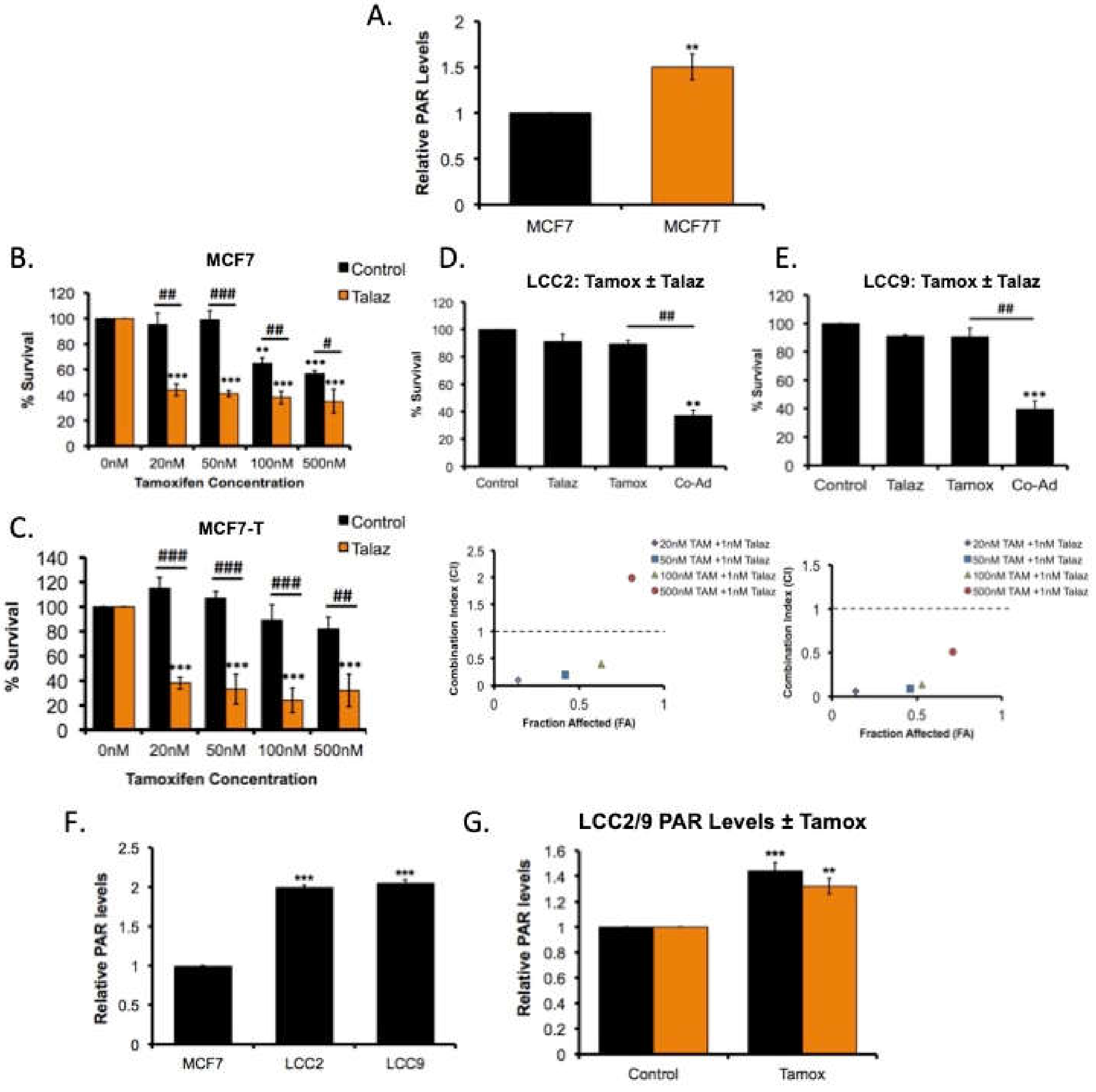

Figure S1. Tamoxifen-talazoparib co-administration decrease cell survival and increase PARP activity. (**A**) To measure basal PAR levels MCF7 and MCF7-T cells were subjected to PAR ELISA. (**B**) MCF7 and (**C**) MCF7-T cells were treated with increasing concentrations of tamoxifen (x-axis) with and without talazoparib (Talaz) and subjected to colony formation assays. Data depicted is a representation of raw data used to construct Figure 1F. 0 nM treatment contains neither tamoxifen

nor talazoparib. (**D**) LCC2 and (**E**) LCC9 ER+ tamoxifen resistant breast cancer cells were treated (72 h) with 1 μM tamoxifen (Tamox) or 1nM talazoparib (Talaz) alone or in combination. Treated cells were subjected to colony formation assay (top) or synergy determination using the Chou-Talalay method (bottom). (**F**) To measure basal PAR levels, LCC2 and LCC9 cells were subjected PAR ELISA. **(G)** LCC2 and LCC9 cells were treated (24 h) with 100 nM Tamox. Treated cells were subjected to PAR ELISA. Results are representative of at least three independent experiments ± SEM. * $p < 0.01$, ** $p < 0.001$, *** $p < 0.0001$ compared to control, # $p < 0.01$, ## $p < 0.001$, ### $p < 0.0001$ relative to bracketed treatment.

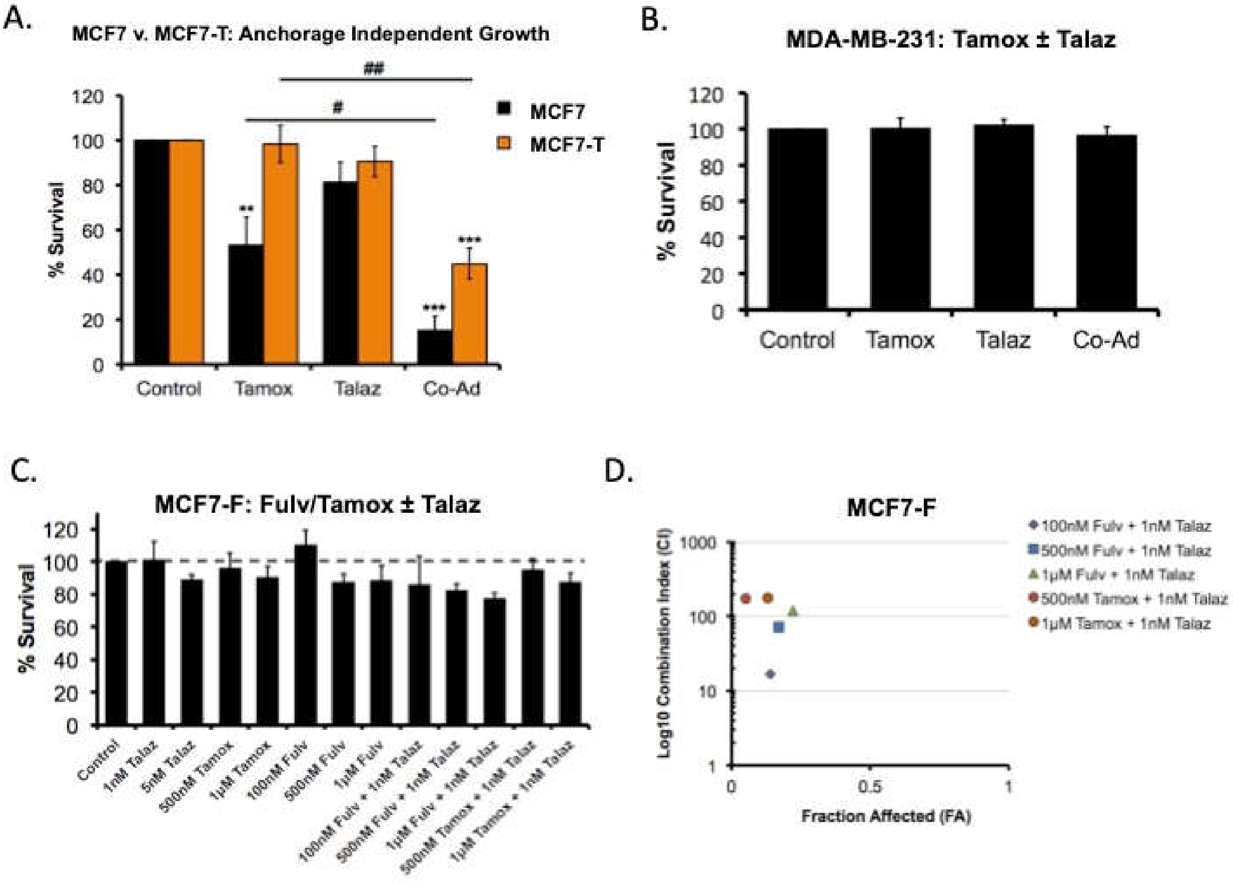

Figure S2. Tamoxifen-talazoparib efficacy is ERα-dependent. (**A**) MCF7 and MCF7-T cells were treated (72 h) with 100 nM Tamox in the presence and absence of 1 nM Talaz. Treated cells were subjected to anchorage-independent growth assays. (**B**) MDA-MB-231 breast cancer cells were treated (72 h) with 1 μM tamoxifen (Tamox) or 1nM talazoparib (Talaz) alone or in combination. Treated cells were subjected to colony formation assay. MCF7-F (ER-) cells were treated for 72 h with indicated concentrations of the anti-estrogens Tamox or fulvestrant (Fulv) alone or in combination with Talaz. Post treatment, cells were subjected to either (**C**) colony formation assays or (**D**) synergy determination using the Chou-Talalay method. Dotted line denotes 100% survival. Results are representative of at least three independent experiments ± SEM. * $p < 0.01$, ** $p < 0.001$, *** $p < 0.0001$ compared to control, # $p < 0.01$, ## $p < 0.001$ relative to bracketed treatment.

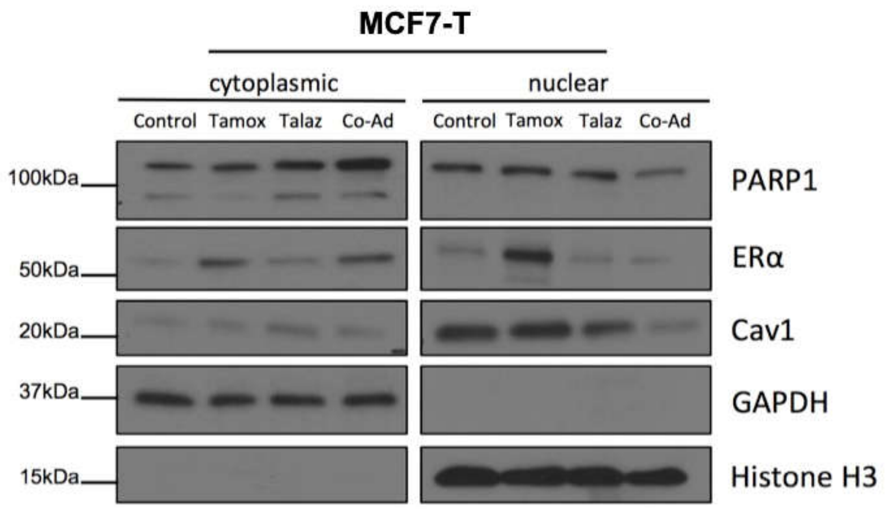

Figure S3. Tamoxifen and talazoparib alter ERα nuclear localization. MCF7-T cells were treated for 4 h with 100 nM tamoxifen (Tamox) alone or in combination with 1 nM talazoparib (Talaz, pre-treat 24 h). Post treatment cells were subjected to cellular fractionation and western blot analysis performed against the indicated antibodies. Blot shown is representative of three separate experiments.

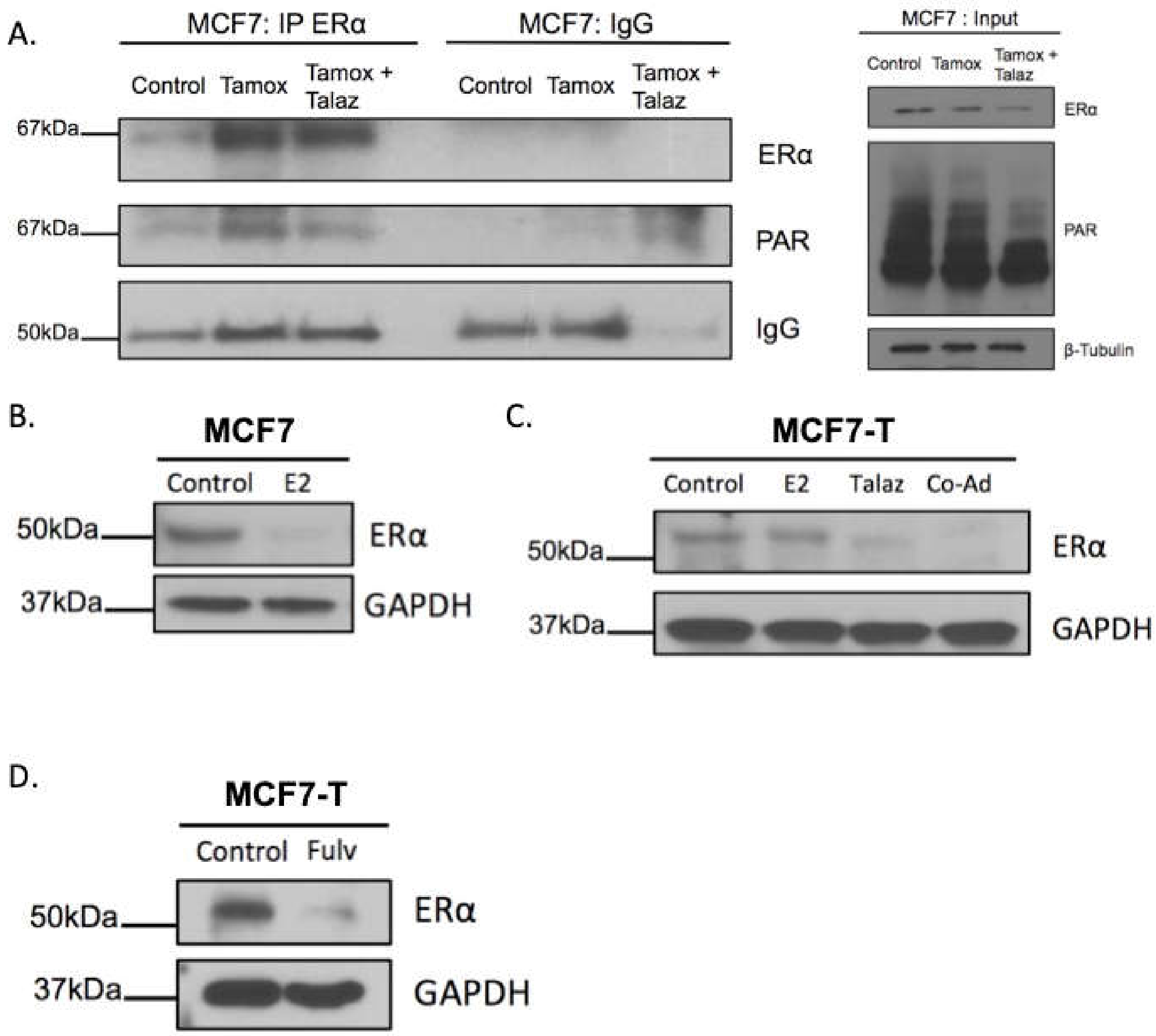

Figure S4. Tamoxifen induces ER PARylation in tamoxifen-sensitive MCF7 cell line. (**A**) MCF7 cells were treated for 4 h with 100 nM tamoxifen (Tamox) or 1 nM talazoparib (pre-treat 24 h, Talaz) alone or in combination. Treated cells were subjected immunoprecipitation (IP) against ER*α* and the indicated antibodies. (**B**) MCF7 cells were treated for 8 h with 10 nM estradiol (E2) and subjected to western blot analysis. (**C**) MCF7-T cells were treated with E2 (8 h, 10 nM) in the presence and absence of Talaz (pre-treat 24 h, 1 nM) and subjected to western blot analysis. (**D**) MCF7-T cells were treated for 24 h with 100 nM Fulvestrant (Fulv) and subjected to western blot analysis. Blots shown are representative of at least three separate experiments.

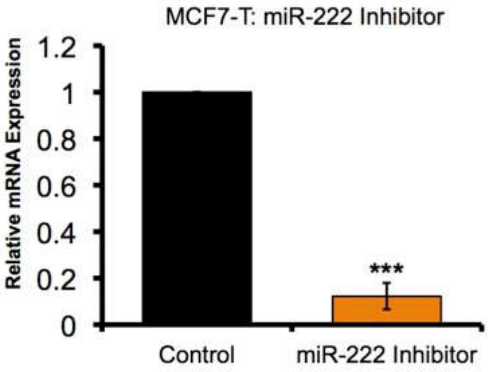

Figure S5. miR-222 inhibition decreases expression of miR-222. MCF7-T cells were treated for 24 h with miR-222 inhibitor and subjected to qRT-PCR analysis to measure miR-222 expression. Results are representative of at least three independent experiments ± SEM. *** $p < 0.0001$.

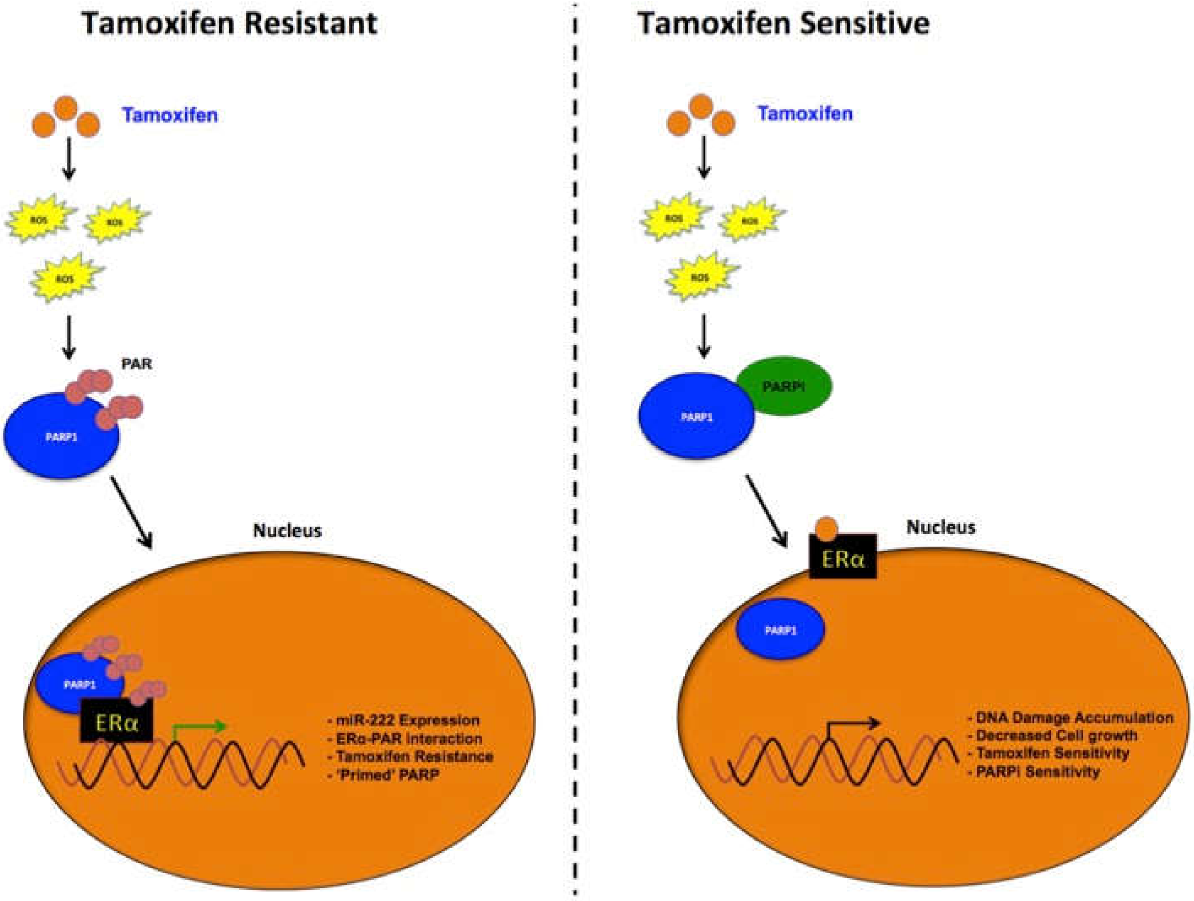

Figure S6. Proposed model: (Left; Tamoxifen Resistance) Tamox increases cellular oxidative damage. Increased oxidative damage promotes increased miR-222 expression, PARP activation and ERα-PAR interaction, mediating Tamox response. Additionally, Tamox-mediated miR-222 expression 'primes' the cell towards PARPi sensitivity. (Right, Tamoxifen Sensitivity) Tamox-Talaz co-administration increases DNA damage accumulation and decreases both ERα localization to ERα-target genes and ERα PARylation. Decreased ERα PARylation contributes to increased Tamox sensitivity, in an ERα-dependent manner.
